# Growth Factor Priming Differentially Modulates Components of the Extracellular Matrix Proteome in Chondrocytes and Synovium-Derived Stem Cells

**DOI:** 10.1371/journal.pone.0088053

**Published:** 2014-02-07

**Authors:** Elena Alegre-Aguarón, Sonal R. Sampat, Jennifer C. Xiong, Ryan M. Colligan, J. Chloë Bulinski, James L. Cook, Gerard A. Ateshian, Lewis M. Brown, Clark T. Hung

**Affiliations:** 1 Department of Biomedical Engineering, Columbia University, New York, New York, United States of America; 2 Quantitative Proteomics Center, Columbia University, New York, New York, United States of America; 3 Department of Biological Sciences, Columbia University, New York, New York, United States of America; 4 Comparative Orthopaedic Laboratory, University of Missouri, Columbia, Missouri, United States of America; 5 Department of Mechanical Engineering, Columbia University, New York, New York, United States of America; University of Pittsburgh, United States of America

## Abstract

To make progress in cartilage repair it is essential to optimize protocols for two-dimensional cell expansion. Chondrocytes and SDSCs are promising cell sources for cartilage repair. We previously observed that priming with a specific growth factor cocktail (1 ng/mL transforming growth factor-β1, 5 ng/mL basic fibroblast growth factor, and 10 ng/mL platelet-derived growth factor-BB) in two-dimensional culture, led to significant improvement in mechanical and biochemical properties of synovium-derived stem cell (SDSC)-seeded constructs. The current study assessed the effect of growth factor priming on the proteome of canine chondrocytes and SDSCs. In particular, growth factor priming modulated the proteins associated with the extracellular matrix in two-dimensional cultures of chondrocytes and SDSCs, inducing a partial dedifferentiation of chondrocytes (most proteins associated with cartilage were down-regulated in primed chondrocytes) and a partial differentiation of SDSCs (some collagen-related proteins were up-regulated in primed SDSCs). However, when chondrocytes and SDSCs were grown in pellet culture, growth factor-primed cells maintained their chondrogenic potential with respect to glycosaminoglycan and collagen production. In conclusion, the strength of the label-free proteomics technique is that it allows for the determination of changes in components of the extracellular matrix proteome in chondrocytes and SDSCs in response to growth factor priming, which could help in future tissue engineering strategies.

## Introduction

Adult articular cartilage has a very limited ability for natural repair following injury, which has led to intense research toward the development of cell-based therapies for cartilage repair [1]. Autologous chondrocyte implantation [2] is one of several current strategies for cartilage repair. This technique requires surgical invasion of normal articular cartilage. However, repair of large cartilage defects is difficult due to the limited proliferative potential of chondrocytes, a low number of healthy chondrocytes in damaged cartilage, and donor-site morbidity [3].

To overcome these limitations, regenerative medicine strategies using mesenchymal stem cells (MSCs) are being developed. MSCs have the ability to differentiate *in vitro* into multiple cell types, including chondrocytes, adipocytes, and osteocytes [4], [5]. MSCs are positive for some surface markers: CD73 (SH3/4), CD90 (Thy-1), and CD105 (endoglin), and negative for most hematopoietic lineage markers [6]. The clinical potential of MSCs as a cell source for cartilage tissue engineering has been extensively studied [7]–[9]. Many *in vitro* strategies have been utilized to induce chondrogenic differentiation, including incubating with chemically defined culture media [10], supplementing with selective growth factor(s) [11], and co-culturing with mature chondrocytes [12].

MSCs can be isolated from several sources, including bone marrow, adipose tissue, periosteum, and synovium [5], [13]–[16]. Of these MSC sources, the synovium is a particularly attractive source of stem cells not only because of its superior chondrogenic capacity [17], but also because it is easily regenerated after arthroscopic harvesting [18]. Synovium-derived stem cells (SDSCs) have potential for both *in vitro* cartilage tissue engineering [3], [18] and *in vivo* cartilage regeneration [19], [20]. Several groups have shown the multi-differentiation potential of SDSCs, thus confirming the mesenchymal potential of SDSCs [5], [21], [22]. With appropriate stimulation, SDSCs are capable of migrating into articular cartilage defects and differentiating toward chondrocytes [23]. SDSCs may be a tissue-specific stem cell for tissue regeneration, as they are capable of responding most aptly to signaling in the joint, thus fostering cartilage tissue regeneration [16].

Our laboratory has shown that both chondrocytes and SDSCs are promising cell sources for cartilage repair [3], [24]. Cartilage tissues grown with chondrocytes have led to engineered cartilage with superior mechanical properties, compared to tissues engineered using stem cells, providing the primary motivation for the chondrocyte cell source adopted in this study. As a result, parallel studies were carried out using canine chondrocytes and SDSCs. Adult canine cells were utilized since the dog represents an important large preclinical animal model for musculoskeletal research [25].

In order to create functional tissue, we used a growth factor expansion protocol that we have observed to be efficacious for expanding bovine chondrocytes and SDSCs in producing subsequent functional tissue matrix. Cell passaging and concurrent priming with chemical factors are often necessary steps in cell-based strategies for regenerative medicine [26]. The fact that physiological formation of articular cartilage occurs through the combination of several growth factors, and that both cartilage and synovium originate from a common group of mesenchymal precursor cells, suggests that cartilage formation utilizing SDSCs occurs similarly [27], [28]. The growth factor cocktail used to prime both chondrocytes and SDSCs in this study consisted of transforming growth factor beta 1 (TGF-β1), basic fibroblast growth factor (bFGF), and platelet-derived growth factor-BB (PDGF-BB) [29]. Previously, this growth factor cocktail was shown to increase the proliferation rates and maintain the chondrogenic potential of human articular chondrocytes [30].

To understand the effects of using this growth factor cocktail on canine chondrocytes and SDSCs, we undertook comparative proteomics analysis. This technique is potentially powerful for this effort, since it quantifies differences in expression of proteins among different biological states. It also allows for the detection of proteins with post-translational modifications; this information is not provided by genomic analyses. Recently, proteomics approaches were applied to studies with MSCs [31]–[33].

The objective of this study was to use comparative proteomics to investigate the impact of growth factor priming on two-dimensional (2D) canine chondrocyte and SDSC cultures, by identifying differentially regulated cartilage proteins. Cells cultured without growth factor supplementation served as the control. Based on our previous SDSC work [3], we hypothesized that primed cells in 2D culture would differentially express some extracellular matrix (ECM) proteins associated with cartilage.

## Materials and Methods

### Ethics Statement

The dog tissue used in the studies was from waste (discarded) tissues from dogs euthanized for other purposes and are therefore IACUC exempt. The healthy, control tissue was obtained from University of Missouri (Manuscript author: James L. Cook) after being euthanized for other purposes. The study “Canine Models for Diagnosis and Treatment of Cartilage Pathology”, that produced the original canine work, was approved by IACUC from the University of Missouri-Protocol Number 3447.

### Tissue Harvesting and Cell Expansion

**Figure 1:**
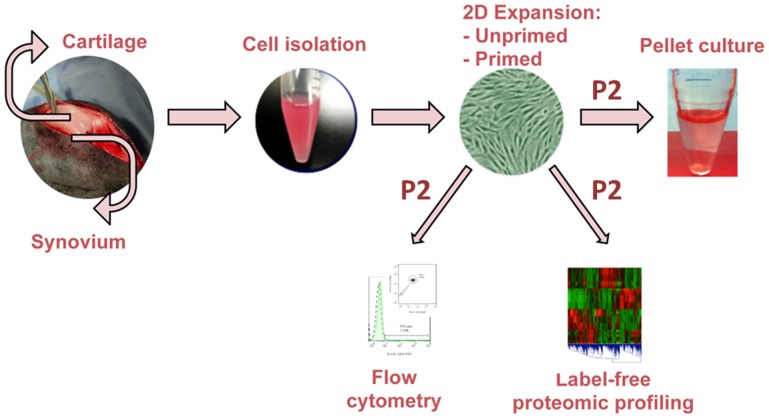
Schematic of experiments. Cells isolated from canine cartilage and synovium were cultured in 2D with (primed, -P) or without (unprimed, -U) a growth factor cocktail (see Materials and Methods). Surface marker expression by flow cytometry and label-free proteomic profiling were assessed in chondrocytes and SDSCs at passage 2 (P2). Cells were subsequently evaluated for chondrogenic capacity in 3D pellet culture at passage 2 (P2).

Cartilage and synovial tissue were harvested from adult knee joints from dogs euthanized for other purposes. Typically, both hind limbs were harvested from each animal and pooled together for the study. Cartilage and synovium were digested using type IV collagenase (310 U/mg, Worthington) in medium containing 10% (v/v) fetal bovine serum (FBS) (Atlanta Biologicals) at 37°C in a humidified 5% CO_2_ atmosphere. Digested cells were filtered through a 70 µm porous mesh and remaining tissue was discarded. Chondrocytes were cultured in high-glucose Dulbecco’s modified Eagle’s medium (hgDMEM, GIBCO) at a density of 22×10^3^ cells/cm^2^ and SDSCs in alpha-minimum essential medium (α-MEM, GIBCO) at a density of 1.76×10^3^ cells/cm^2^, both with 10% (v/v) FBS and 1% (v/v) antibiotic-antimycotic (100 U/mL penicillin, 100 µg/mL streptomycin and amphotericin B) (PSAM, from GIBCO), denoted as passage 1 (P1). To determine the effects of priming cells towards a chondrogenic lineage in 2D culture, culture media was supplemented with a cocktail of growth factors (1 ng/mL TGF-β1, 5 ng/mL bFGF, and 10 ng/mL PDGF-BB for the ‘primed’ group) [3], [25], [34]. Cells expanded without the cocktail of growth factors served as the control or ‘unprimed’ group. When cells reached 80–90% confluence, they were detached from the flask with 0.05% trypsin/0.53 mM EDTA (Mediatech) and replated at the same initial density (P2). When plated cells reached 80–90% confluence at P2, chondrocytes and SDSCs (unprimed and primed) were harvested for flow cytometry and proteomics analyses (Fig. 1). Cultures were fed three times per week. Proliferation rate was calculated at each passage using the doubling time formula: t_d_ = t_c_ × ln(2)/ln(final/initial), where: t_d_ = doubling time (days), t_c_ = time in culture (days), final = number of cells harvested, initial = number of cells initially seeded.

### Flow Cytometry

At P2, chondrocytes and SDSCs were resuspended in phosphate-buffered saline (PBS, GIBCO) containing 2 mM EDTA (GIBCO) and fluorescein isothiocyanate (FITC)- or phycoerythrin (PE)-conjugated monoclonal antibodies. Unstained cells were used as a fluorescence negative control. Antibodies against CD31 (Abcam), CD34 (Abcam), and CD45 (Invitrogen) were used as negative indicators. CD31 (PECAM-1) is an endothelial cell marker; CD34 and CD45 (LCA) are hematopoietic cell markers. Antibodies against MSC markers, CD105 (endoglin, Abcam), CD151 (PETA-3, BD Pharmigen), and CD166 (ALCAM, BioLegend) were also used. Cells were incubated in the dark at room temperature for 15 minutes, after which they were washed and resuspended in 0.5 mL PBS containing 2 mM EDTA. Cell fluorescence was evaluated by flow cytometry using a FACSCalibur flow cytometer (Becton Dickinson). The resulting data were analyzed by FlowJo software (version 9.3.2).

### Proteomics Analyses

A label-free protein profiling technique for mass spectrometry-based shotgun proteomics was performed using a NanoAcquity liquid chromatograph and a Synapt G2 HDMS QTOF mass spectrometer (Waters Corp.). Cells were washed with ice cold PBS, lysed in 0.3% SDS, TRIS-buffered saline with 1% Protease Inhibitor Cocktail (Sigma-Aldrich), precipitated using a methanol/chloroform extraction and then dissolved in 0.1% RapiGest™ SF detergent-containing (Waters Corp.) 50 mM ammonium bicarbonate. Dithiothreitol was added and the solution was sonicated and boiled for 5 minutes. Protein concentration was determined using the Bradford Protein Assay (Bio-Rad). Cysteines were alkylated with iodoacetamide. Proteins were digested with trypsin and 50 fmol of a digest of yeast alcohol dehydrogenase was added as an internal detection control. The mass spectrometer was equipped for traveling wave ion mobility spectrometry (TWIMS). Ion mobility provided separation on the basis of shape and cross-sectional area, in addition to the conventional mass-to-charge ratio. The use of ion mobility for shotgun proteomics has been demonstrated to be effective in increasing proteome depth of coverage [35], [36]. Three independent biological replicates from three separate cultures were analyzed. For each replicate, three 120 min liquid chromatography (LC)/mass spectrometry (MS) runs were carried out, providing a total of 36 chromatograms (nine replicate analyses for each group) in resolution/ion mobility mode. Spectra were recorded with a 0.6 second scan time, analyzed with ProteinLynx Global Server V.2.5, RC9, (Waters Corp.) on a Lenovo D20 Workstation with Xeon Processor equipped with a NVIDIA Tesla C2050 graphics processing unit (GPU) processor with 448 CUDA cores. Data was searched against an NCBI Refseq database of canine sequences derived from Release 47. This database containing 33,335 canine sequences (18,873,091 residues). Accurate mass and retention time matches of precursors were compared across all LC/MS runs for label-free intensity-based quantitation were performed with Rosetta Elucidator software Ver. 3.3.0.1.SP3_CRE52.21 (Ceiba Solutions, Inc.) as described previously [33]. Statistical analyses were generated by Elucidator with p-values calculated from an application of an error model developed for large-scale microarray data as adapted for proteomics within the Elucidator program. Protein ratio p-values for differential expression were calculated by the Elucidator program using the *xdev* parameter [37], [38].

The combination of label-free MS^E^ data acquisition and post-processing with Elucidator data mining software for accurate mass and retention time matching is validated independently by other groups [35], [39]–[41]. From one C-P sample, all three chromatograms were outliers (derived from this one sample), were excluded from the dataset based as typical on visual inspection [42] and principal component analysis (data not shown). This individual sample may have had some aberrant culture problem or problem with protein extraction. Such exclusions are a normal prerequisite for analysis of proteomics data [42].

In order to better understand the relevance of the resulting canine proteins and for pathway analysis, canine Protein GI numbers were converted into official gene symbols using the DAVID conversion tool found at http://david.abcc.ncifcrf.gov/conversion.jsp. Following this, canine official gene symbols were converted to their human equivalent (when available) using Homologene on NCBI. UniProt [43] was used to generate all of the annotated protein data. Finally, the DAVID tool [44] was used as an interface to the Kyoto Encyclopedia of Genes and Genomes (KEGG) [45] pathway repository at Kyoto University to generate pathway diagrams that illustrate some possible relationships from the proteomics data.

### In vitro Chondrogenic Differentiation

Chondrogenic differentiation of canine chondrocytes and SDSCs at P2 was evaluated in a 28-day micropellet culture study [13]. Briefly, 0.5×10^6^ cells were centrifuged (300×g for 7 min) in 15 mL conical polypropylene tubes (Becton Dickinson) and the resulting pellet was cultured in 0.5 mL of chondrogenic medium (hgDMEM, 1% (v/v) PSAM, 1% (v/v) ITS™+Premix (BD Biosciences), 100 µg/mL sodium pyruvate (Sigma), 50 µg/mL L-proline (Sigma-Aldrich), 0.1 µM dexamethasone (Sigma-Aldrich), with 50 µg/mL ascorbate-2-phosphate (Sigma-Aldrich) and 10 ng/mL TGF-ß3 (R&D) added fresh during each media change. SDSC pellets were additionally treated with 500 ng/mL bone morphogenetic protein-2 (BMP-2, GenScript). All chondrocyte and SDSC pellets were fed twice per week.

### Biochemical Analysis

Time points for biochemical analyses were conducted on days 0, 14 and 28. Pellets were digested in proteinase K solution overnight at 56°C, as previously described [46]. Biochemical analysis was performed to determine the glycosaminoglycan (GAG) content using the dimethylene blue assay [47]. In addition, the orthohydroxyproline assay [48] was used to assess collagen content. Overall collagen content was calculated by assuming a 1∶7.64 orthohydroxyproline-to-collagen mass ratio [49]. The DNA content was determined using the PicoGreen kit (Invitrogen), according to the manufacturer’s standard protocols. GAG and collagen content was normalized to DNA content.

### Statistical Analysis

Statistics were performed using a student’s t-test to compare groups (primed vs. unprimed in both chondrocytes and SDSCs). Results were considered to be significant for p≤0.05. Data was analyzed using GraphPad Prism software (version 4.0c).

## Results

### Cell Proliferation

**Figure 2:**
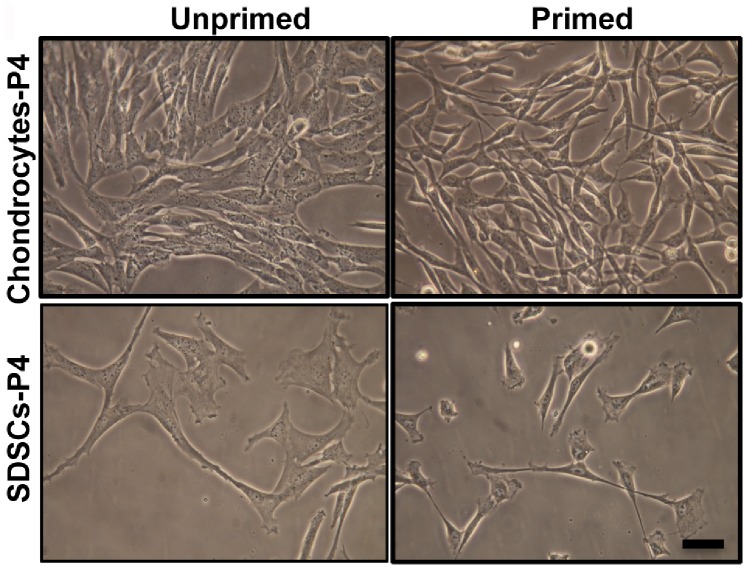
Morphological differences between unprimed and primed cells. Phase contrast micrographs (20×) of canine chondrocytes and SDSCs at passage 4 cultured unprimed (left side) or primed (right side). Scale bar = 50 µm. Initial plating density was 12.5-fold greater for chondrocytes than SDSCs.

Treatment with the growth factor cocktail resulted in changes in morphology and proliferation of cells. Within a few hours of plating, both SDSCs and chondrocytes had adhered to the tissue culture treated plastic. Non-adherent cells were removed from cultures 48 hours after plating by changing the media. At P4, unprimed cells were more widespread over the surface and showed a myoblast-like morphology while primed cells displayed a more elongated, spindle shape (Fig. 2).

**Figure 3:**
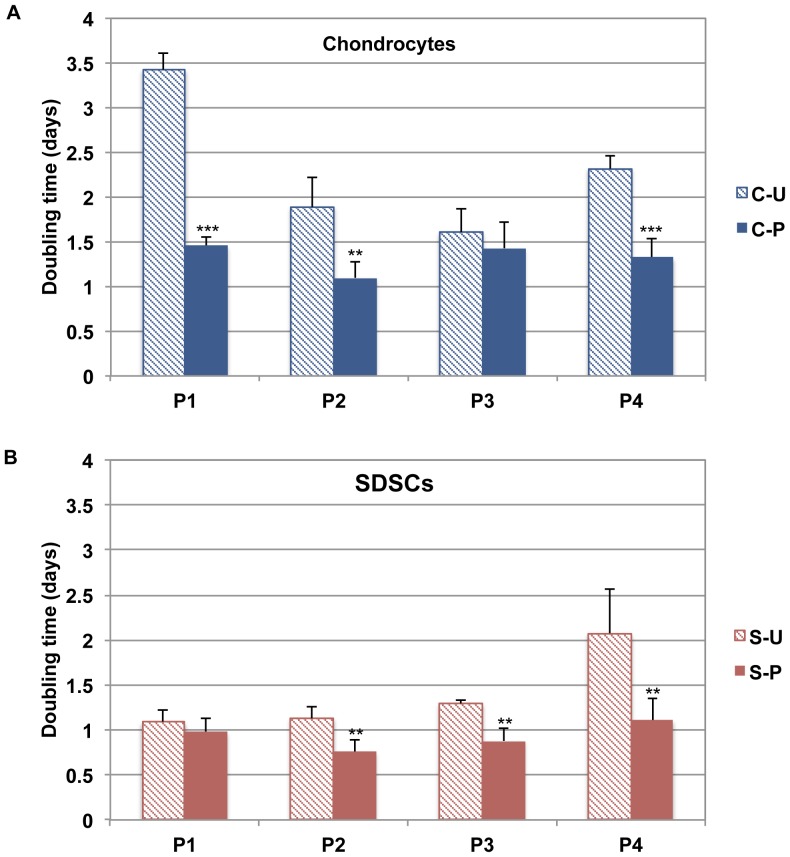
Doubling times of canine chondrocytes and SDSCs. Doubling times of canine (A) chondrocytes (unprimed (C-U), primed (C-P)) and (B) SDSCs (unprimed (S-U), primed (S-P)) from P1 to P4 (n = 5). Statistically significant differences relative to unprimed cells are represented as **p<0.01 and ***p<0.001.

The doubling times from P1 to P4 for each cell line (unprimed chondrocytes: C-U; primed chondrocytes: C-P; unprimed SDSCs: S-U; primed SDSCs: S-P) are shown in Figure 3. For each cell type (chondrocytes and SDSCs), primed cells clearly proliferated more rapidly than unprimed cells, as seen by the doubling rates (chondrocytes: p<0.001 at P1 and P4, p<0.01 at P2; SDSCs: p<0.01 at P2, P3, and P4).

### Flow Cytometry

Cell surface marker expression was analyzed for canine chondrocytes and SDSCs at P2 (Table 1). The expression of CD31 (endothelial cell marker), CD34, and CD45 (hematopoietic cell markers) was <5% for all groups and no differences were detected between the groups. The highest expression of CD105 (a MSC marker) was seen in S-P (79%, p<0.001; S-P vs. S-U). Both primed SDSCs and chondrocytes showed similar expression of CD151 (∼ 60%) (Table 1). Surprisingly, S-U showed the highest expression (82%) of CD151 as compared to its expression in S-P (59%) (p<0.05). However, the lowest expression of CD151 was found in C-U (27%), roughly half of its expression in C-P (58%; p<0.01). Another MSC marker, CD166, was positive (mean ≥ 95%) in SDSCs, regardless of treatment. In chondrocytes, the percentage of CD166-positive cells was higher in C-P (76%) (p<0.01; C-P vs. C-U).

**Table 1:** Phenotype of canine chondrocytes (C-U, C-P) and SDSCs (S-U, S-P) at passage 2.

Expression (%)	C-U	C-P	S-U	S-P
CD31	0.02±0.02	0.04±0.03	0.06±0.07	0.00±0.01
CD34	0.13±0.14	0.12±0.14	0.48±0.46	0.14±0.19
CD45	0.63±0.31	1.20±0.9	4.25±3.09	1.14±0.89
CD105	45.78±9.65	35.18±5.23	61.90±5.46	78.82±3.31***
CD151	26.67±9.76	57.80±3.61**	81.57±9.61	59.00±6.53*
CD166	52.50±5.53	75.83±7.18**	95.00±3.49	96.28±1.29

Percentages of positive cells are shown as mean percentage ± SD (n = 5). Statistically significant differences relative to unprimed cells are represented as *p<0.05, **p<0.01 and ***p<0.001.

### Proteomics Analyses

Proteomic analysis focused on 1,766 proteins from which three or more peptides were detected (see Table S1). Criteria for further protein selection were based on p-value, ratio between –P and –U (considered of interest when the increase or decrease was >1.75-fold), and peptide count, which allowed us to narrow our study to 357 proteins of interest (labeled as * in Columns BL to BM in Table S1). SDSCs had a greater number of proteins of interest, in that 172 proteins were up-regulated (the abundance of these proteins was higher in S-P than in S-U cells), while only 21 were up-regulated by priming in chondrocytes (see Table S1). To gain insight into the biological significance of the altered proteins during the priming treatment, the differentially expressed proteins were categorized according to their reported biological functions. Functional characterization of these 357 proteins of interest reveals that more than 50% are involved in cellular organization (including proteins whose primary function is in the ECM, cytoskeleton, or membrane organization) and transcription, protein synthesis, and turnover (see Table S1).

**Figure 4:**
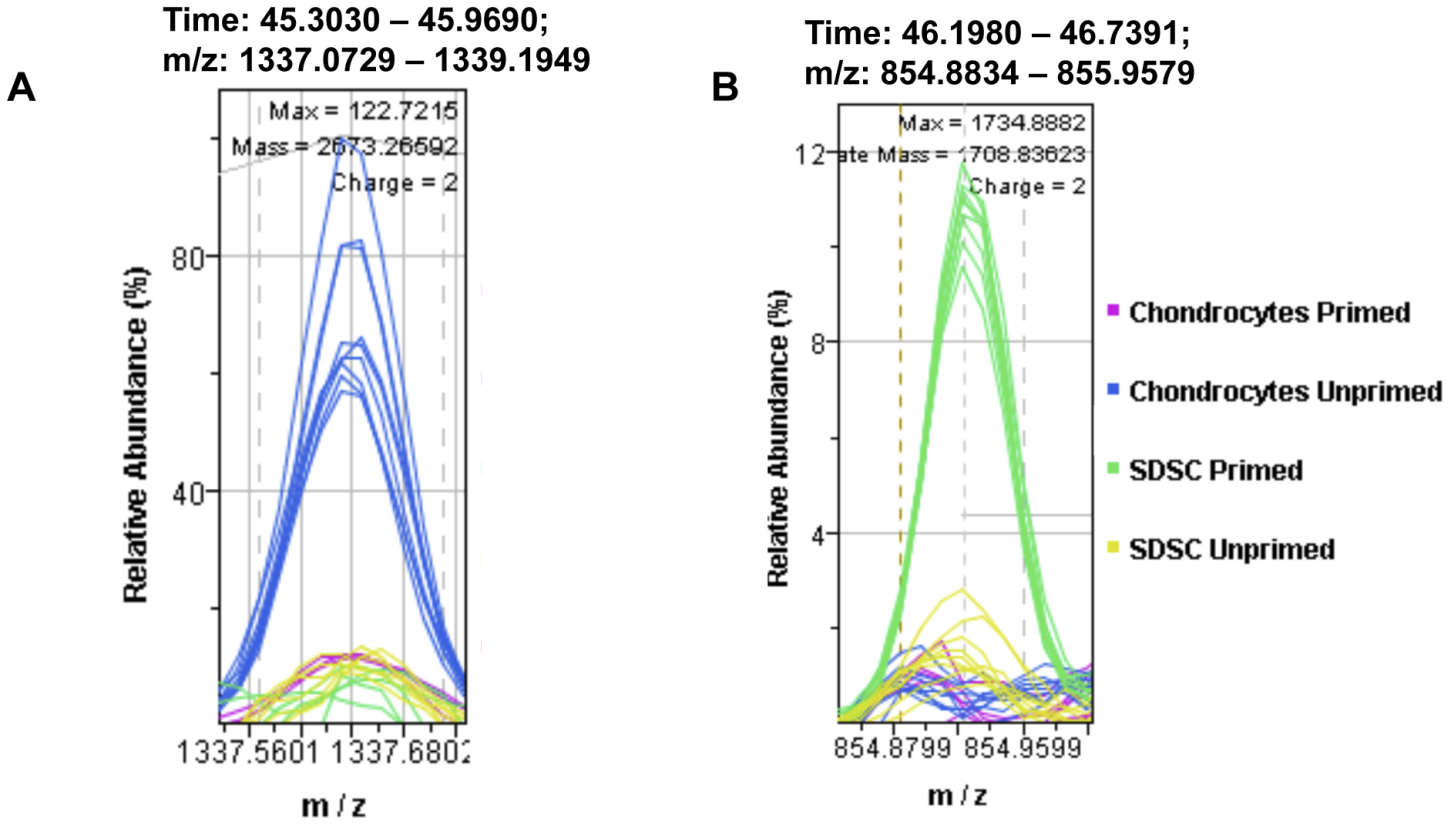
Examples of differential protein expression in canine chondrocytes and SDSCs. A) Example of differential expression of collagen alpha-1(II) chain. The plot represents one isotopic signal from the mass spectrum of peptide GFTGLQGLPGPPGPSGDQGASGPAGPSGPR at 1337.1 m/z and 45.6 min retention time. Unprimed chondrocytes (C-U) cells exhibit strong accumulation of this protein, compared to primed chondrocytes (C-P) samples or either S-U or S-P samples B) Example of differential expression of aminopeptidase N (CD13). Plot represents one isotopic signal from the mass spectrum of peptide ESALLYDPQSSSIGNK at 854.9 m/z and 46.3 min retention time. Primed SDSCs (S-P cells) exhibit strong accumulation of this protein in response to growth factor priming; in comparison, neither C-P nor unprimed SDSCs (S-U cells) show a significant signal.

In primed chondrocytes, some ECM-related proteins were down-regulated (i.e., abundance of these proteins was higher in C-U than C-P). These included type I collagen, type II collagen (4-fold decrease) (Fig. 4A), type V procollagen, type V collagen, type XII collagen, which interacts with type I collagen, and aggrecan (2.77-fold decrease) (Table 2). Thrombospondin 1, a marker of articular cartilage, was less abundant in both primed chondrocytes and SDSCs (1.82-fold decrease) (Table 3). In addition, other proteins involved in synthesis and processing of ECM components were also down-regulated: procollagen-lysine, 2-oxoglutarate 5-dioxygenase 2 isoform b precursor which forms hydroxylysine residues in Xaa-Lys-Gly- sequences in collagens, and lysyl oxidase preproprotein isoform 1, which is responsible for the post-translational oxidative deamination of peptidyl lysine residues in precursors to fibrous collagen and elastin (Table 2). On the other hand, tenascin, another ECM protein expressed in chondrocytes, was up-regulated with priming (1.92-fold increase). Transforming growth factor-beta induced protein IG-H3 precursor, which binds to types I, II, and IV collagens, was highly up-regulated (6.39-fold increase) in C-P too (Table 2). Additional proteins associated with cell stress, rescue or defense, were more abundant in C-U, including heat shock protein beta-1, alpha crystallin B chain, nucleoredoxin, and stress-70 protein (Table 2).

**Table 2:** Most prominent differentially expressed proteins found in the proteomics analysis in chondrocytes.

NCBI GI Number	Protein Name	P-value[C-P vs. C-U]	Ratio[C-P/C-U]	Number of Unique Peptides
304376314	Tenascin	0	1.92	35
164665422	Aggrecan core protein precursor	3.5E-18	0.36	3
74004974	Collagen alpha 2(V) chain precursor	3.2E-17	0.42	22
73991481	Destrin (Actin-depolymerizing factor) (ADF)	9.0E-21	0.51	4
73990680	Procollagen-lysine, 2-oxoglutarate 5-dioxygenase 2 isoform b precursor	1.4E-40	0.52	25
73973310	Alpha 1 type XII collagen short isoform precursor isoform 2	2.2E-21	0.53	92
73971374	Transforming growth factor-beta induced protein IG-H3 precursor (Beta IG-H3) (Kerato-epithelin)(RGD-containing collagen associated protein) (RGD-CAP)	7.0E-39	6.39	5
73970918	Stress-70 protein, mitochondrial precursor (75 kDa glucose regulated protein) (GRP 75)(Peptide-binding protein 74) (PBP74) (Mortalin) (MOT) isoform 21	1.4E-18	0.46	3
73970573	Lysyl oxidase preproprotein isoform 1	0	0.32	8
73967750	Procollagen, type V, alpha 1	6.1E-43	0.43	23
73967327	Nucleoredoxin	1.2E-21	0.56	5
73958530	Mitogen activated protein kinase 3	9.9E-35	0.46	3
57085977	Alpha crystallin B chain (Alpha(B)-crystallin) (Rosenthal fiber component)(Heat-shock protein beta-5) (HspB5) isoform 1	0	0.1	5
55742776	Collagen alpha-1(II) chain	0	0.25	10
50979116	Heat shock protein beta-1	0	0.38	8
50978940	Collagen alpha-2(I) chain precursor	0	0.53	52
50978774	Collagen alpha-1(I) chain precursor	4.5E-38	0.48	85

Most of the protein names are truncated from the full names listed in the database for simplicity.

**Table 3:** Most prominent differentially expressed proteins found in the proteomics analysis in chondrocytes and SDSCs.

NCBI GI Number	Additional NCBI GI Numbers	Protein Name	P-value[C-P vs. C-U]	P-value[S-P vs. S-U]	Ratio[C-P/C-U]	Ratio[S-P/S-U]	Number ofUnique Peptides
73999965		Thrombospondin 1 precursor	0	0	0.55	0.55	45
73992265		Endothelial protein C receptor precursor (Endothelial cell protein C receptor) (Activatedprotein C receptor) (APC receptor) (CD201 antigen)	0	1.1E-41	6.04	2.69	4
73981286		D-3-phosphoglycerate dehydrogenase (3-PGDH)	7.7E-38	0	0.54	1.98	16
73954763	73954765	Dihydrolipoamide S-acetyltransferase (E2 component of pyruvate dehydrogenasecomplex) isoform 2	3.1E-18	0	2.01	2.94	3

Most of the protein names are truncated from the full names listed in the database for simplicity.

In SDSCs, some transcription factors and protein products linked to chondrogenic differentiation were down-regulated. Y-box transcription factor, known to be a regulator of collagen translation, and collagen alpha 1(III) chain precursor isoform 2, present in most soft connective tissues along with type I collagen, were down-regulated. In contrast, procollagen-lysine, 2-oxoglutarate 5-dioxygenase 1 precursor (Lysyl hydroxylase 1) (LH1) isoform 3, which forms hydroxylysine residues in nascent collagen chains, and type VI collagen, were both up-regulated in S-P cells. Meanwhile, other ECM proteins, namely biglycan and lumican, were down-regulated in S-P (3.45- and 2.7-fold decrease, respectively). Integrin beta 1, a receptor for collagen and fibronectin, and integrin alpha-5 precursor, a receptor for fibronectin and fibrinogen, were both down-regulated (Table 4).

**Table 4:** Most prominent differentially expressed proteins found in the proteomics analysis in SDSCs.

NCBI GI Number	Protein Name	P-value[S-P vs. S-U]	Ratio[S-P/S-U]	Number ofUnique Peptides
225637546	Aminopeptidase N	0	4.50	9
158819069	Prohibitin	1.3E-41	1.80	12
74004777	Collagen alpha 1(III) chain precursor isoform 2	0	0.54	19
74001592	Collagen, type VI, alpha 1 precursor	0	3.17	11
73996304	Integrin alpha-5 precursor (Fibronectin receptor alpha subunit) (Integrin alpha-F) (VLA-5) (CD49e)	0	0.47	31
73978407	Protein disulfide-isomerase A4 precursor (Protein ERp-72) (ERp72) isoform 3	0	2.02	21
73971652	Gelsolin precursor (Actin-depolymerizing factor) (ADF) (Brevin) (AGEL) isoform 4	0	3.31	6
73968271	CD63 antigen (Melanoma-associated antigen ME491) (Lysosome-associated 1membrane glycoprotein 3)(LAMP-3) (Ocular melanoma-associated antigen) (OMA81H) (Granulophysin) (Tetraspanin-30) (Tspan-30)	0	2.34	7
73964198	C-1-tetrahydrofolate synthase, cytoplasmic (C1-THF synthase)	0	2.01	28
73963026	Cofilin 2	0	2.06	7
73954721	Transgelin isoform 2	2.1E-30	0.35	3
73950968	Nuclease sensitive element binding protein 1 (Y-box binding protein-1) (Y-box transcription factor) (YB-1)(CCAAT-binding transcription factor I subunit A) (CBF-A) (Enhancer factor I subunit A) (EFI-A)(DNA-binding protein B) (DBPB)… isoform	0	0.46	24
73950916	Procollagen-lysine,2-oxoglutarate 5-dioxygenase 1 precursor (Lysyl hydroxylase 1) (LH1) isoform 3	0	2.24	3
73948624	Integrin beta 1 isoform 1D precursor	9.3E-29	0.46	25
57097203	Lumican precursor (Keratan sulfate proteoglycan lumican) (KSPG lumican)	2.3E-31	0.37	18
50979010	Biglycan precursor	1.3E-31	0.29	21

Most of the protein names are truncated from the full names listed in the database for simplicity.

In general, our data suggest that S-P were metabolically more active than S-U and most of the proteins whose level increases metabolism and cellular energy were up-regulated. For example, ATP synthases, in NAD, lipid, or carbohydrate biosynthetic enzymes, as well as oxidative tricarboxylic cycle enzymes (see Fig. S1), and mitochondrial respiratory chain components were all up-regulated (see Table S1). Moreover, some enzymes involved in glycine biosynthesis and collagen synthesis (D-3-phosphoglycerate dehydrogenase, C-1-tetrahydrofolate synthase), were also up-regulated (Table 3, 4). Protein disulfide-isomerase A4 precursor (Protein ERp-72) (ERp72) isoform 3, that catalyzes the rearrangement of -S-S- bonds in proteins and related to collagen biosynthesis and assembly too, was also up-regulated (2.02-fold increase) (Table 4). A number of proteins involved in proteasome-dependent protein turnover (see Fig. S2), or amino acid turnover (see Fig. S3), were increased in primed cells.

Proteomic data also documented the altered abundance of proteins whose function it is to regulate the cell cycle or cell fate. Endothelial protein C receptor (stem cell marker) was up-regulated in both C-P (6.04-fold increase) and S-P (2.69-fold increase) (Table 3). A marker typically used to identify stem cells, aminopeptidase N (CD13), was 4.5-fold up-regulated in S-P (Table 4) (Fig. 4B). CD63, a tetraspanin, was also up-regulated (2.34-fold increase) (Table 4). As stated previously, S-P cells showed greater proliferation than S-U cells and proteins associated with cell cycle, cell division, and DNA replication were mostly up-regulated (Table S1).

A variety of proteins known to regulate the cytoskeleton, subcellular organization, or the mechanics of secretion, were regulated by priming. Therefore, the majority of proteins associated with the cytoskeleton (actin, microtubules, actin binding proteins (tropomyosin, drebrin, and stathmin that regulate microtubule remodeling), and myosin and dynactin involved in cellular and organelle motility and secretion) were down-regulated (see Table S1). Other actin-remodeling proteins were differentially expressed, including transgelin (down-regulated) or cofilin and gelsolin (up-regulated) (Table 4).

### Biochemical Analysis

**Figure 5:**
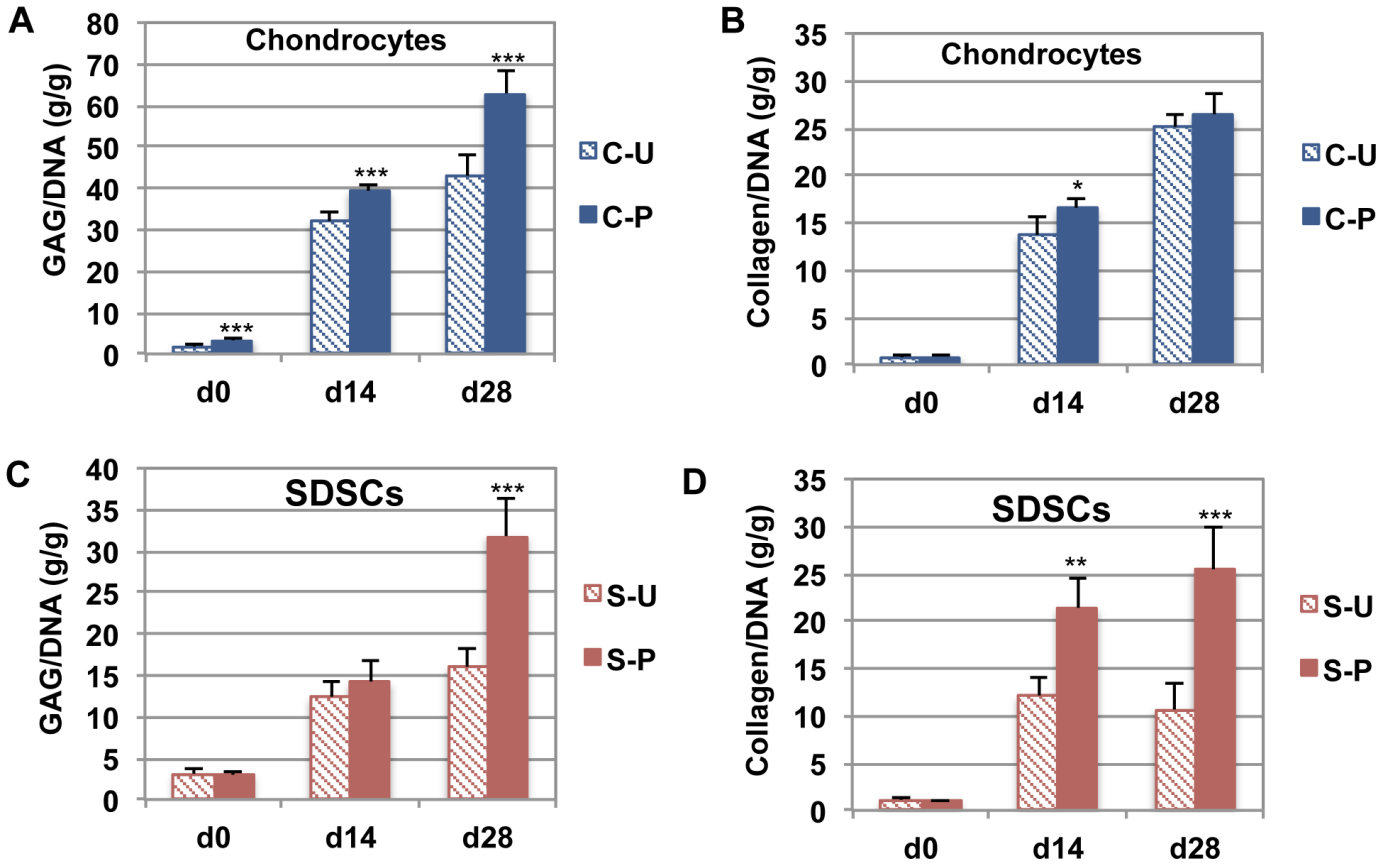
Biochemical properties of canine chondrocyte- and SDSC-pellet culture. (A, C) Glycosaminoglycan (GAG) content (g) normalized to DNA (g). After 28 days in culture, primed cells produced significantly more GAG/DNA. (B, D) Collagen content (g) normalized to DNA (g). Primed SDSCs (S-P cells) produced more collagen after 14 or 28 days. Results are shown as mean ± SD (n = 5). Statistically significant differences relative to unprimed cells are represented as *p<0.05, **p<0.01 and ***p<0.001.

After 28 days in culture, chondrocyte pellets showed significant differences in GAG content (p<0.001), with 62.95±5.69 GAG/DNA in C-P pellets and 43.07±5.19 GAG/DNA in C-U pellets (Fig. 5A). In contrast, C-P pellets showed a higher collagen content than C-U pellets at day 14 (p<0.05), but a similar level by day 28 for both C-P and C-U cells (Fig. 5B). A significant increase in GAG and collagen content was detected in S-P pellets (31.82±4.39 GAG/DNA and 25.39±4.41 collagen/DNA) compared to S-U pellets (15.92±2.24 GAG/DNA and 10.67±2.82 collagen/DNA) (p<0.001) at day 28 (Fig. 5C, D), while no significant differences in DNA content were found over culture time or between groups (data not shown).

## Discussion

The aim of this study was to prime canine chondrocytes and SDSCs with a cocktail of growth factors that increases their potential for chondrogenic differentiation, and to identify differentially regulated cartilage proteins. The results from this study could potentially be used to identify predictors of cells’ utility in cartilage tissue engineering protocols. The current study demonstrated that growth factor priming had a much greater effect on SDSCs than on chondrocytes, as seen by the number of proteins differentially expressed in 2D between primed and unprimed cells, and the differences in GAG and collagen content in 3D pellet culture. Primed SDSCs appear to retain their stem cell potential, although some collagen-related proteins, indicative of differentiation, were up-regulated, which could be significant in their future tendency for chondrogenic differentiation. In contrast, chondrocytes, which start out in a differentiated state, dedifferentiated over the culture period in 2D. Moreover, a dramatic increase in cell number (doubling time), especially in SDSCs, was observed. After 4 weeks of incubation, cells in pellet cultures adopted a chondrocytic fate; GAG and collagen content increased in a time-dependent manner. Recent studies have found that cultured chondrocytes show better chondrogenic differentiation ability than human MSCs [50], [51]. These studies are consistent with our results that chondrocytes produced more GAG than SDSCs in pellet culture.

Although SDSCs are distinct from bone marrow-derived MSCs, they are similar in their surface epitope expression [21]. To date, no epitope unique to only SDSCs has been identified [21]. However, since SDSCs have been characterized as MSCs, surface epitopes specific to MSCs are used for analysis. Of the surface markers expressed by SDSCs, CD105 binds TGF-β1 and TGF-β3 with high affinity. Thus, CD105 may be a required mediator of TGF-β signaling during chondrogenic differentiation of MSCs [52]. Further, it was found that the CD105-positive subpopulation of MSCs from human synovial membrane differentiated toward chondrocyte-like cells [53]. In our study, this marker was more highly expressed in S-P than S-U, and S-P cells differentiated towards chondrocytes more fully than S-U cells, based on their increased GAG and collagen content in pellet culture. CD166, another MSC marker [54], was positive in both S-U and S-P cells, although its expression was significantly higher in C-P than C-U, which may be attributable to the observed dedifferentiation of the chondrocytes.

*In vitro* culture conditions are important in determining the proteome of cells. For example, culturing bone marrow-derived stem cells with bFGF prolonged the cells’ differentiation potential [55]. In agreement with Solchaga et al., who showed that human bone marrow-derived MSCs expanded in the presence of bFGF were smaller in size and faster growing than cells without bFGF supplementation [56], both canine primed SDSCs and chondrocytes showed spindly morphology and proliferated more rapidly.

The first proteomic analysis of human bone marrow-derived MSCs used 2D gels to identify differentially expressed proteins by mass spectrometry [57]. In recent years several key technological developments have significantly advanced both proteomic characterization and biomarker discovery [31]. Since the initial report by Colter et al., proteomics has been used to identify biomarkers implicated in cell migration in diverse tissues including bone marrow, umbilical cord blood, and placenta [58]. In our study, identification of differentially expressed proteins was based on label-free shotgun mass spectrometry and ion mobility spectrometry, which together enhance peptide resolution, and provide orthogonal separation of peptides in the gas phase to increase detection and sequence coverage of proteins. The label-free protein profiling approach has been utilized previously in our laboratory to characterize human adipose-derived stem cells [33]. However, our current study presents the first proteomic profile of SDSCs upon growth factor cocktail stimulation.

Park et al. summarized the proteome identified for human mesenchymal stem cells [59]. It is noteworthy that among the proteins Park et al. identified, some were also identified as proteins of interest in our SDSC study, such as prohibitin, type VI collagen, and CD63, which were all up-regulated in S-P cells. Senescent cells down-regulate prohibitin expression [59]. Thus, S-U cells showed a lower abundance of this protein compared to S-P cells, which could be attributed to their reduced proliferation. Moreover, another stem cell surface marker (CD63) we identified in SDSCs is also expressed on human articular chondrocytes [60] and human marrow stromal cells [61]. Another protein we identified as being up-regulated in S-P has been reported to be a stem cell surface marker, CD13 [54]. However, we found that other proteins previously identified in the MSC proteome, such as transgelin, integrin beta 1, LIM and SH3 domain protein 1, peptidylprolyl isomerase A and lumican [59], were down-regulated in SDSCs (see Table S1).

The presence of smooth muscle proteins was previously demonstrated in bone marrow-derived MSCs [57] and adipose-derived MSCs [62]. However, several isoforms of tropomyosin and α–tubulin were down-regulated in S-P cells (see Table S1). Small leucine-rich proteins, such as decorin, fibromodulin, lumican, and biglycan, which interact with collagen fibrils and influence their fibrillar architecture and function [63], were mostly unchanged, but two of these, lumican and biglycan, were down-regulated in S-P cells. Cartilage oligomeric matrix protein (COMP), a large pentameric glycoprotein and a member of the thrombospondin group of extracellular proteins, also binds collagen and is thought to mediate cell–matrix interactions [64], [65]. Although we did not identify COMP in this study, we did identify a COMP-related protein, protein disulfide isomerase, which functions in the processing and transport of wild-type COMP, in chondrocytes [66]. In addition, protein disulfide isomerase helps in the folding of many other extracellular proteins; thus it was more abundant in S-P cells, where it acts as a subunit for prolyl 4-hydroxylase in collagen biosynthesis, and as a molecular chaperone for assembly of procollagen [67].

*In situ*, articular chondrocytes reside in a hypoxic environment and therefore rely primarily on anaerobic glycolysis to generate ATP [68]. Our study found few differences in proteins involved in energy production between C-P and C-U samples, potentially due to the fact that the culture environment we used was not hypoxic. However, one exception was dihydrolipoamide S-acetyl transferase, a component of the pyruvate dehydrogenase complex (one of the links between glycolysis and the Krebs cycle) [69]. This enzyme was up-regulated in both C-P and S-P cells. In fact, most of the differentially expressed proteins involved in cellular metabolism and energy production were up-regulated in S-P cells.

αB-crystallin (heat-shock protein b5) is a member of the small heat-shock protein family and it functions to protect cells against stress factors, such as heat shock, oxidative stress, osmotic shock, and chemical stress. A decrease of αB-crystallin was associated with a decrease in collagen type II and aggrecan; a decrease in both of the latter typifies dedifferentiation of chondrocytes [70]. Our results appear similar: we found αB-crystallin, type II collagen, and aggrecan were all down-regulated in C-P. Another small heat shock protein, heat shock protein beta 1, was down-regulated in C-P cells, similar to decreases that had been reported for this protein after stimulation with TGF-β in human bone marrow-derived MSCs [31].

As observed with other MSC types [59], the global proteome of SDSCs would be a more reliable predictor of chondrogenic potential than focusing only on specific markers. Thus, it was shown by proteomic profiling that the use of TGF-β1, bFGF, and PDGF-BB to stimulate canine chondrocytes and SDSCs in 2D culture is accompanied by remodeling of the cytoskeleton and other proteins associated with the ECM, and led to better chondrogenic differentiation in 3D culture. Consequently, understanding the importance of multiple proteins within the protein profile obtained by priming may elucidate the underlying mechanism by which this growth factor cocktail mediates better chondrogenesis. Specifically, most proteins associated with cartilage were down-regulated in chondrocytes including collagens, aggrecan, thrombospondin 1, D-3-phosphoglycerate dehydrogenase, procollagen-lysine, 2-oxoglutarate 5-dioxygenase 2 isoform b precursor, and lysyl oxidase preproprotein isoform 1. However, some collagen-related proteins were up-regulated in SDSCs, including type VI collagen, protein disulfide isomerase, D-3-phosphoglycerate dehydrogenase, and C-1 tetrahydrofolate synthase. These results argue that priming mediated a partial dedifferentiation of chondrocytes and a partial differentiation of SDSCs as seen by the regulation of a series of ECM related proteins, both of which allowed expansion of cells that have great chondrogenic potential once placed in 3D culture. That is, ECM components of the priming-induced protein profile may be directly linked with the higher GAG and collagen production demonstrated in pellet culture, both in this study and in constructs seeded with primed SDSCs in previous work [3]. The results of the present study also verify that the label-free profiling proteomics technique is a robust, consistent, and automated technology that is suitable for high throughput quantitative proteomics studies. Future insights in this field will allow for the identification of biomarkers that could produce better tissue engineered cartilage.

### Supporting Information

Proteins detected in this study with three or more peptides are listed in the spreadsheet Table S1. This table includes protein name, NCBI GI number, additional NCBI GI numbers matching same peptides, canine gene, corresponding human gene, human protein name, human keywords, human subcellular location, human gene ontology (GO), human pathway, human catalytic activity, human function, human tissue specificity, intensity, p-values, ratio between primed and unprimed, peptide count, and functional characterization of the 357 proteins differentially expressed in this study. All raw mass spectrometry data files will be uploaded and freely accessible to the global scientific community at the community-supported Chorus project website (http://chorusproject.org).

Supporting Data Figures S1, S2 and S3 were derived by searching differentially expressed proteins in the DAVID and KEGG databases. This material is available free of charge via the Internet at http://www.plosone.org.

## Supporting Information

Figure S1 **Citrate cycle diagram by KEGG.** Some enzymes that were identified as protein of interest from the proteomics data and participate in citrate cycle were up-regulated in SDSCs (represented as red arrows).
(TIF)

Figure S2 **Proteasome diagram by KEGG.** Some proteasomal proteins that were identified of interest from the proteomics data were up-regulated in SDSCs (represented as red arrows).
(TIF)

Figure S3 **Valine, leucine, and isoleucine degradation diagram by KEGG.** Some enzymes that were identified of interest from the proteomics data and participate in valine, leucine, and isoleucine degradation were up-regulated in SDSCs (represented as red arrows).
(TIF)

Table S1 **Differentially Expressed Proteins.** This table includes protein name, NCBI GI number, additional NCBI GI numbers matching same peptides, canine gene, corresponding human gene, human protein name, human keywords, human subcellular location, human gene ontology (GO), human pathway, human catalytic activity, human function, human tissue specificity, intensity, p-values, ratio between primed and unprimed, peptide count, and functional characterization of the 357 proteins differentially expressed in this study.
(XLS)
