# Supplementary figures and images for: Growth Factor Priming Differentially Modulates Components of the Extracellular Matrix Proteome in Chondrocytes and Synovium-Derived Stem Cells
Source: PLoS One. 2014 Feb 7;9(2):e88053. doi: 10.1371/journal.pone.0088053 (PMC3917883; doi:10.1371/journal.pone.0088053)

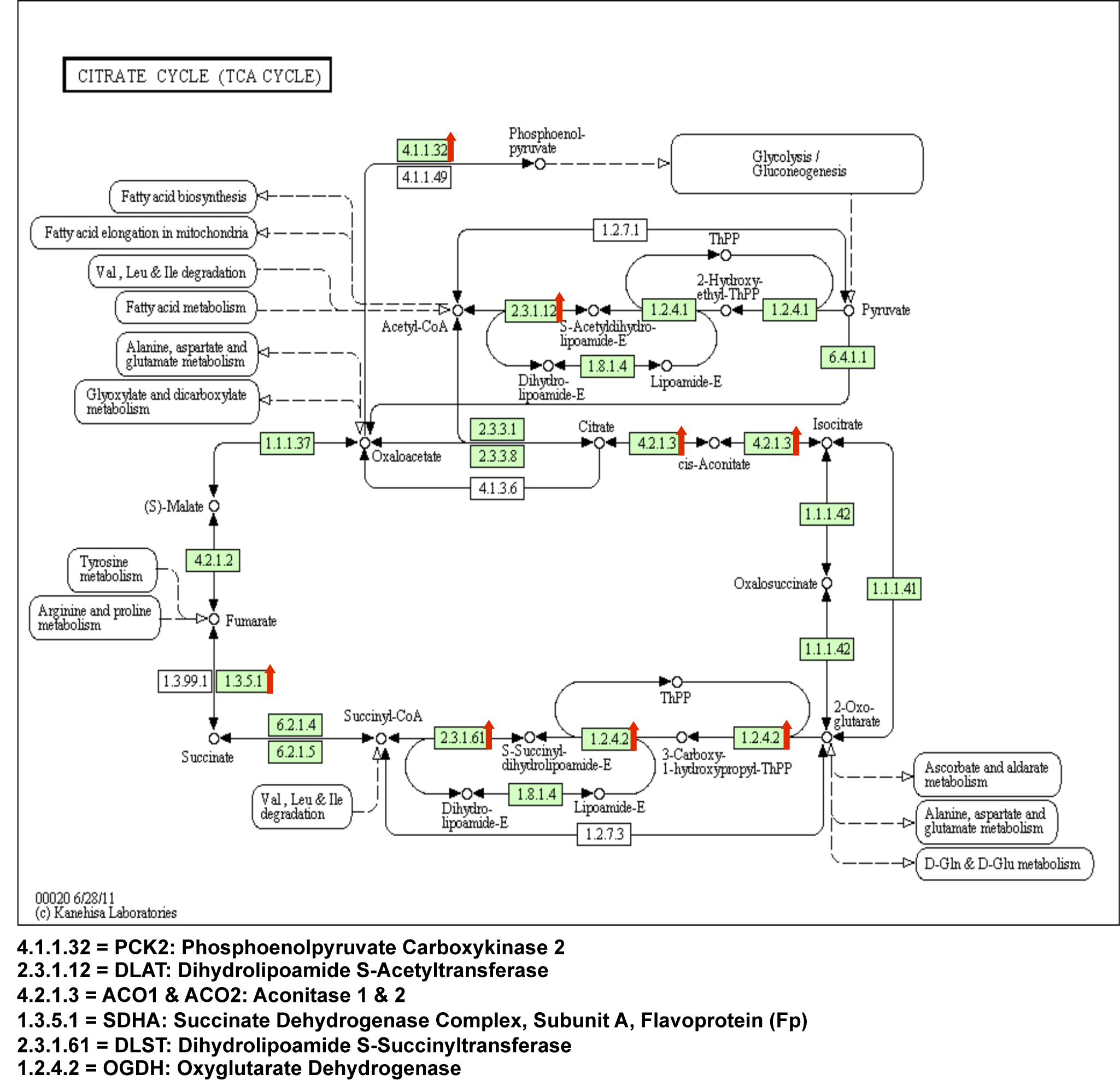

Supplement: Figure S1 — Citrate cycle diagram by KEGG. Some enzymes that were identified as protein of interest from the proteomics data and participate in citrate cycle were up-regulated in SDSCs (represented as red arrows). (TIF) [file pone.0088053.s001.tif]

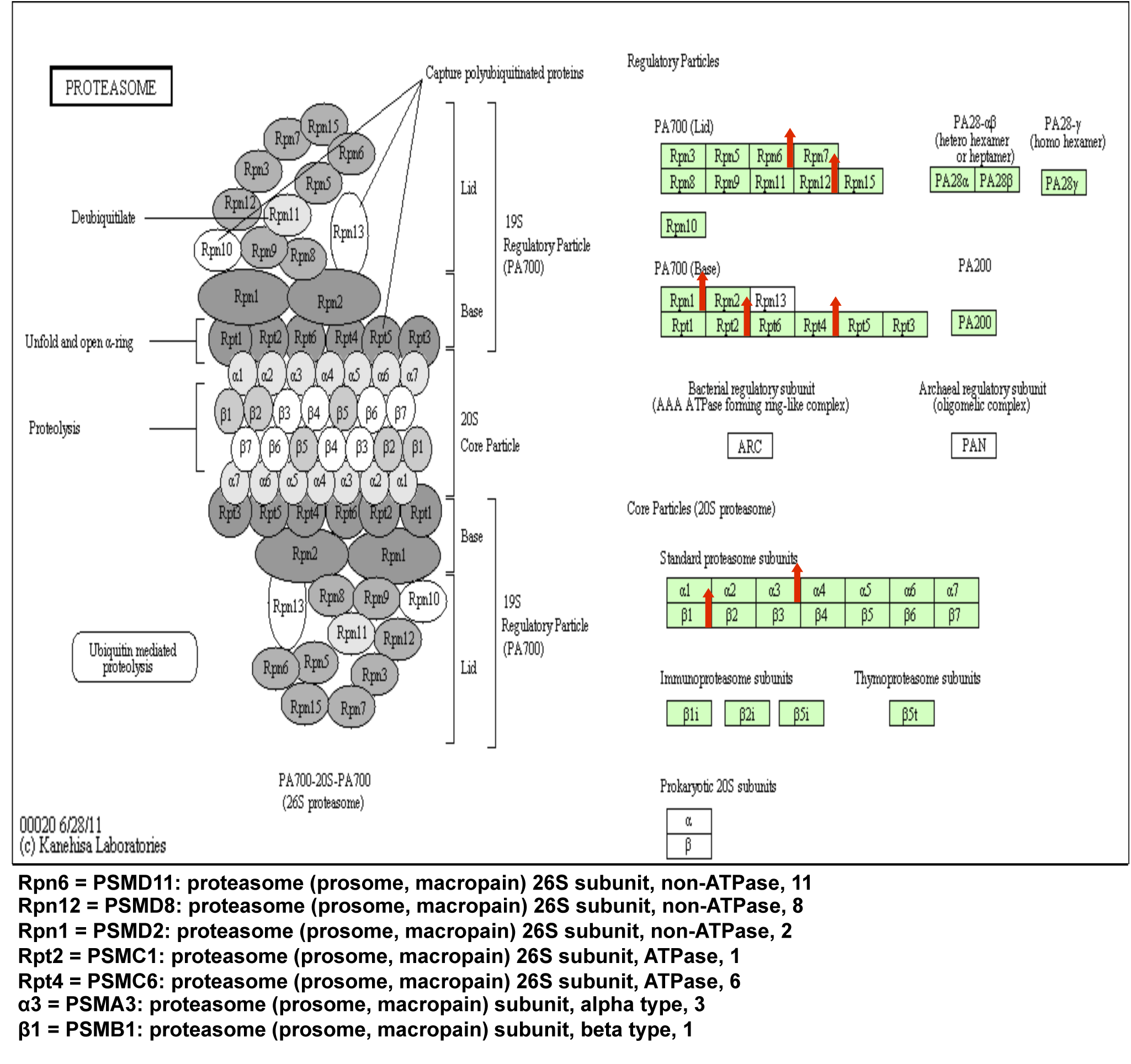

Supplement: Figure S2 — Proteasome diagram by KEGG. Some proteasomal proteins that were identified of interest from the proteomics data were up-regulated in SDSCs (represented as red arrows). (TIF) [file pone.0088053.s002.tif]

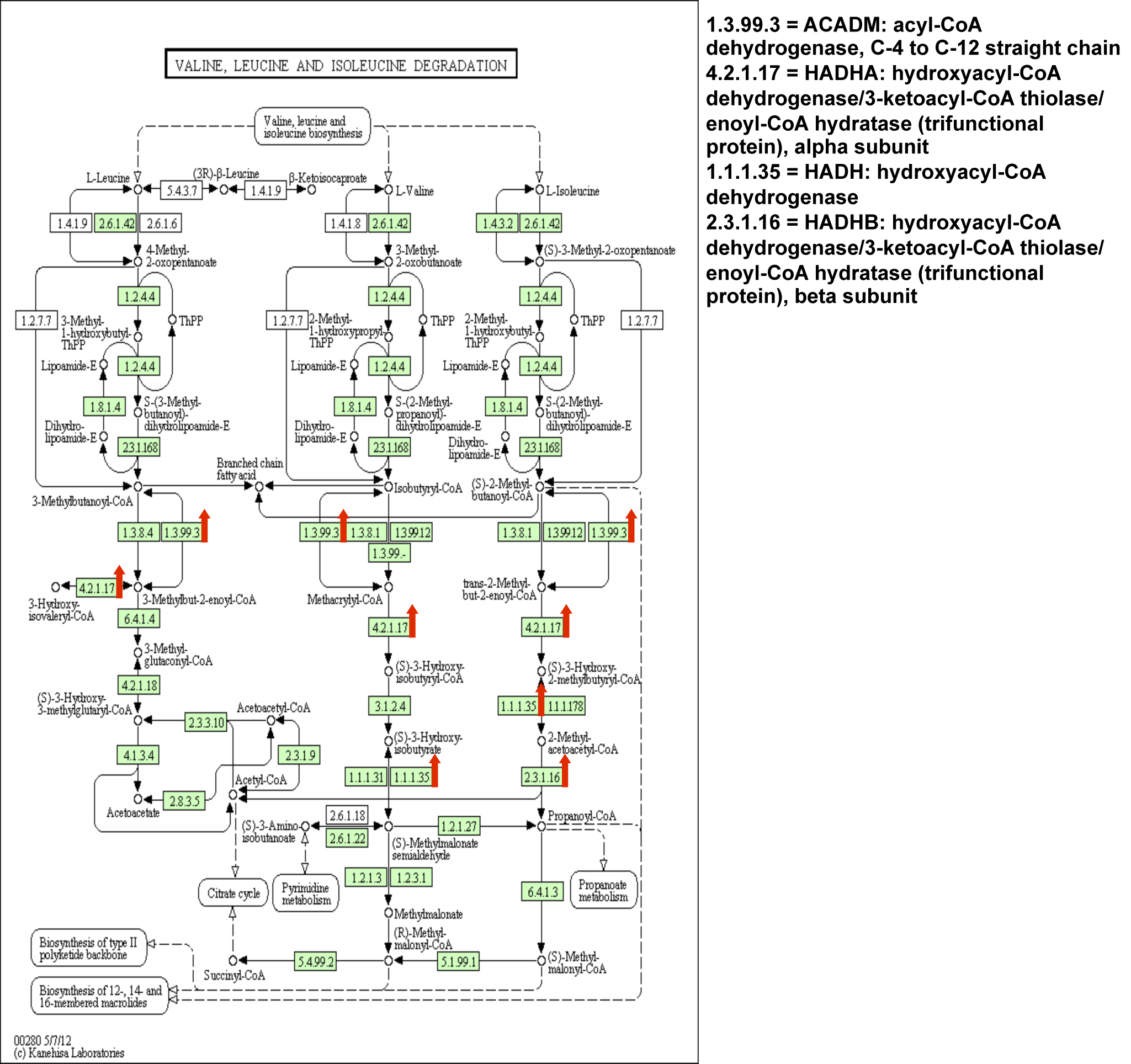

Supplement: Figure S3 — Valine, leucine, and isoleucine degradation diagram by KEGG. Some enzymes that were identified of interest from the proteomics data and participate in valine, leucine, and isoleucine degradation were up-regulated in SDSCs (represented as red arrows). (TIF) [file pone.0088053.s003.tif]
